# Supplementary material for: Are carbon emissions trading and green financial instruments synergistic? -Comprehensive quantitative research based on content analysis
Source: PLoS One. 2024 Mar 7;19(3):e0298601. doi: 10.1371/journal.pone.0298601 (PMC10919601; doi:10.1371/journal.pone.0298601)
Supplement: S1 Data — (ZIP) [file pone.0298601.s001.zip › Data/地级市Z_F工作报告.zip!/║┌┴·╜¡/─╡╡ñ╜¡╩╨/└┤╫╘╣1⁄2╓┌║┼í╛┬φ┐╦╩2╛▌═°í┐_2014.docx]

各位代表：

　　我代表市人民政府，向大会作工作报告，请予审议，并请市政协委员提出意见。

　　一、2013年工作回顾

　　过去一年，在市委正确领导下，在人大、政协监督支持下，我们紧紧依靠全市人民，求实创新、跨越争先，全面推进“五城建设”，全市经济社会保持快速发展的良好势头。

　　——经济发展保持高位增长。积极克服经济下行压力，主要指标在全省实现保位争先。预计地区生产总值突破1200亿元、社会消费品零售总额突破400亿元，分别增长12.5%和15%，增速居全省前列；固定资产投资预计超过1000亿元、增长30%，公共财政预算收入完成91.3亿元、增长15.1%，增速高于全省平均水平；进出口总额与去年基本持平，保持全省首位；城镇居民人均可支配收入预计达到19200元、增长15.8%；农民人均纯收入预计达到14900元、增长15%，总量实现全省11连冠。

　　——产业项目建设持续升温。三年产业项目攻坚完成投资1065亿元，推进500万元以上项目2345个、省级重点项目130个，全市固定资产投资连续七年保持30%以上增长。2013年开复工产业项目1031个，完成投资449.4亿元，产业投资占固定资产投资比重达到57.1%。园区发展空间不断拓展、载体功能全面提升，全市重点园区新增面积4.7平方公里，形成17平方公里储备用地；市开发区晋升国家级经济技术开发区，新开工产业项目78个、完成投资13.5亿元、增长59%，“一个孵化中心、五个加速器”项目孵化体系初步形成；林口开发区、宁安工业园、阳明工业示范基地、大庆路新型工业园区晋升为省级园区(基地)。招商融资取得新突破，全市新引进亿元以上项目105个，其中产业项目75个，引进域外资金870.8亿元、增长34.4%；全面完成政府性融资任务，域内金融机构存贷比提高5个百分点，域外金融机构贷款余额达到85亿元。

　　——沿边开放向纵深推进。外贸转型升级取得新进展，境外园区新增面积4.4平方公里、入驻企业达到62户，地产品出口比重、进口资源落地加工比重分别达到12%和47%，我市成为国家级蔬菜外贸转型升级示范基地、省级石油装备外贸转型升级示范基地。华晟物流海关直接监管公用型保税仓库投入运营，获得允许外籍船舶承运等政策支持，中俄陆海联运大通道向常态化迈出新步伐。在俄举办“牡丹江活动周”，签署人文交流、经贸合作项目57个。商贸会展有新提升，牡达等3个专业市场投入运营，绿地等6个商贸综合体开工建设，我市被确定为全省唯一地市级国家肉菜流通追溯体系建设试点；打造牡绥会展经济圈，举办第六届中国(牡丹江)—俄罗斯(远东)国际木业博览会、中国绥芬河国际口岸贸易博览会、东宁宝玉石文化节、全省首届非物质文化遗产博览会等展会23个。旅游产业加快发展，镜泊小镇、莲花新镇等25个重点旅游项目完成投资21.6亿元，新增4A级景区4个；开展了镜泊湖秋冬旅游文化节等冬春旅游活动，开通了海参崴、济州岛航线；获批异地办照政策，跨境旅游便利度进一步提升；我市被纳入“美丽中国之旅”推广计划，雪乡荣膺“中国十大最美乡村”，全市接待境内外游客人数和旅游业总收入分别增长21.8%和24.5%。

　　——精品特色农业不断壮大。粮食生产再创新高，总产达到63亿斤。绿色特色农业进一步壮大，国家级大型绿色食品原料基地发展到430万亩，新增菌棚室5792栋、食用菌达到37.1亿袋(块)，新增蔬菜棚室8500栋、果菜达到64.2万亩，白瓜籽达到50.2万亩，新建养殖小区(大场)49个，林下经济产值增长26%。农业产业化、市场化、品牌化步伐加快，12个产业化项目竣工投产，全市规模以上龙头企业达到175户，农民专业合作社发展到1396个；绿色食品电子商务中心投入运营，国家级牡丹江木耳批发市场交易量占全国2/3；穆棱肉牛、东宁大米、宁安虹鳟鱼通过国家地理标志认证，响水、皓月等11户企业产品被评为“首届黑龙江消费者最喜爱的100种绿色食品”。全市育林造林20.4万亩，治理水土流失36万亩，田间作业综合机械化率达到90%，新增宁安、穆棱两个省级农业科技园区。

　　——工业经济快速发展。从政策、机制、服务三个环节入手抓运行、稳增长，预计规模以上工业增加值实现230亿元、增长19%，居全省第二位。实施支柱企业产能翻番和中小企业成长工程，友搏、啤酒等产能翻番项目竣工投产，页岩油中试基地、桦林佳通半钢子午胎等项目加快推进，亿丰煤气、富通空调通过改制改造实现扩张发展，工业固定资产投资预计增长35%，净增规模以上工业企业43户。组建了十大产业技术创新战略联盟和十大科技创新服务平台，高新技术产业产值增长25%，我市首获全国科技进步先进市。产业集群建设有新发展，石油钻采装备产业成为省级创新型产业集群建设试点，装备制造、林纸等支柱产业占全市规模以上工业比重达到79.7%。县域经济主要指标保持全省前列，省政府在穆棱、海林召开现场会推广我市县域经济发展经验。

　　——城镇化建设取得突破性进展。加速“1核5城18镇100区”新型城镇化体系建设，城关镇和重点镇完成棚户区改造拆迁200万平方米，建成“幸福家园”小区16个、5000多户农民喜迁新居，积极推进响水“两化”项目和温春新镇建设，与森工农垦新建续建合作项目52个、完成投资16.6亿元。对18个重点镇实行强镇扩权试点，集体林权主体改革全面完成，全市土地流转面积达到201.5万亩。稳步推进新农村建设，在54个试点社区推广穆棱保安新村模式，全市有4个五星级村、26个四星级村通过省级评定，36个村启动美丽乡村建设，国家级和省级生态镇村达到214个。

　　——城市面貌发生显著变化。启动城市总规修改和空间发展战略、城市综合交通体系等25个专项规划编制，实施了城市地下管网普查和数字牡丹江地理信息框架系统建设。政府主导市区城建投资达到98.8亿元，老城改造“五项重点工程”全面完成，拓宽改造光华街等6条主干路，升级改造新安街等3条重点街路，翻建整修44条便民道路，重点维修市区道路200条，东四跨江桥二期通车，青梅大桥主跨合拢，牡海城际公路开工建设。棚户区改造向城郊乡镇延伸，实施百万平方米棚户区改造，开工建设公租房2500套、廉租房200套，改造农危房1.5万户。加快生态园林建设，完成“三溪一河”治理收尾和主城区江道环境综合整治，“十大公园”、“九大广场游园”全部建成，市区新增绿地50万平方米；新建、改造燃气管网14.3公里，新增集中供热面积60万平方米。实施城市管理“363”工程，推进公安、城管联勤执法，开展清雪抗灾专项行动，城市环境和秩序不断优化。高度重视人大一号议案办理，完成百万平方米老旧小区庭院整治，41个小区面貌焕然一新。

　　——民生质量明显改善。全市民生支出110亿元，占公共财政预算支出50%，30件利民实事和保障民生各项工作全面完成年度任务。就业社保进一步加强。发放小额创业贷款1.6亿元，新开发公益性岗位2228个，新增城镇就业7.2万人，城镇登记失业率控制在3.3%以内。切实加强社会保障，企业离退休人员人均月增资241元，实现“城居保”、“新农保”全覆盖；工伤人员伤残津贴提高18%，城镇居民医保年补助标准提高到280元，新农合重特大疾病保障病种增加到20种、报销封顶线提高到8万元；城乡低保标准分别提高到月人均382元和年人均2181元；募集志愿爱心帮扶基金2900万元，“两节”期间投入资金947万元补贴困难群众，发放廉租住房补贴4380万元。

　　社会事业全面发展。公共文化设施网络实现城乡全覆盖，我市成为全国首批、全省唯一公共文化服务体系示范区，唐渤海国上京龙泉府遗址被确定为国家考古遗址公园。基层公办医疗机构全部实施国家基本药物制度，国家级示范社区卫生服务中心建设走在全省前列，市二院等4所医院投入使用，我市成为省级卫生城市；以学区制为重点的义务教育均衡发展经验在全省推广，改造了65所标准化学校和公办幼儿园，市一中成为全国普通高中多样化、特色化试点品牌学校，宁安、穆棱成为国家现代农村职业教育改革试点县。新增农民健身工程、全民健身路径130处，我市成为全国青少年校园足球试点城市。

　　社会管理进一步加强。推进社区建设，新建城乡达标社区56个，其中精品示范社区18个；深入开展“安全生产责任落实年”和食品药品安全专项整治活动；实施源头治访、依法治访，化解重大疑难信访积案33件，全市信访案件和集体访分别下降17.1%和5.5%；深入开展“三大行动”，加强社会治安综合治理，刑事、治安案件分别下降30.8%和34.4%，全市保持安定和谐的良好局面。

　　——政府行政效能进一步提升。调整市区财政管理体制，优化开发区管理体制，启动新一轮行政审批制度改革，进一步优化28个重点部门165个审批事项办事流程。第三次全国经济普查进展顺利。务实开展“接地气、连民心”服务群众教育实践活动，通过“市民开放日”活动，推出各类惠民举措540项，解决群众诉求和生产生活问题。完善目标管理和大督查机制，对19个部门管理职能进行调整，推进政府工作提速提效。实施“六五”普法规划，开展“阳光司法”，我市成为全国法治城市。认真办理人大代表建议、批评和意见265件，政协委员提案374件，解决了一批群众关心的热点难点问题。按照反“四风”要求，压缩“三公经费”和行政事业性项目支出5%，开展了办公用房、公务用车、楼堂馆所专项整治。在重点部门建立权力运行程序公开制度、开展廉政风险防控工作，对政府投资项目实施跟踪审计，严厉查处违纪违法案件，政府部门总体保持了良好的工作状态。

　　统计、外事侨务、机关事务、老龄、气象、地震、档案、人防、边防、民兵预备役等工作得到加强，民族宗教、人口计生、妇女儿童、红十字、残联等工作取得新进展。

　　各位代表，过去一年成绩来之不易，这是市委总揽全局、正确领导的结果，是人大、政协有力监督、帮助支持的结果，是全市上下同心同德、团结奋进的结果。在此，我代表市人民政府向全市人民，向人大代表、政协委员，向离退休老领导、老同志，向各民主党派、工商联、无党派人士、人民团体，向各位企业家、科技工作者，向驻牡部队和武警官兵，向驻牡中省直单位，向所有关心支持牡丹江发展的各界人士，表示衷心地感谢和崇高地敬意!

　　回顾一年工作，虽然主要指标增速在全省领先，处于高位增长区间，但受宏观经济下行影响，全市地区生产总值、固定资产投资、进出口总额等指标增速没有完成年初的预期目标。经济社会发展还存在不容忽视的问题：立市型大项目、产业项目少，工业支撑能力弱，外贸对地方经济的拉动作用不强，生态旅游、中心城市等优势还没有充分发挥；城市基础设施老化陈旧，物业管理水平有待提高；环境治理压力大，大气质量亟待改善；群众对干部队伍中存在的“四风”问题还有很多意见，发展环境有待进一步优化。对此，我们要采取有力措施，在不断解决突出矛盾和问题中推进跨越发展。

　　二、2014年目标任务

　　今年是全面深化改革的开局之年，是完成“十二五”目标的关键之年。全市经济社会发展的总体要求是：深入贯彻党的十八届三中全会精神和省委、省政府决策，深入实施“五大规划”，按照市委十一届五次全会部署，坚持稳中求进、求快、求好，坚持用全面深化改革统揽全局，以实施产业攻坚推进新发展，以提升沿边开放构筑新优势，以加快城镇化催生新动力，以发展民生事业集聚新能量，以转变政府职能增添新活力，沿着“五城建设”方向迈出求实创新、跨越争先的更大步伐。

　　经济社会发展主要预期目标是：地区生产总值增长11%，固定资产投资增长30%，公共财政预算收入增长13%，规模以上工业增加值增长16%，社会消费品零售总额增长14%，进出口总额增长10%，城乡居民收入增长13%，万元GDP综合能耗下降3.7%，完成四种主要污染物减排任务。

　　重点抓好九个方面工作：

　　(一)开展新一轮产业项目攻坚，增强投资拉动推进新发展。强化“全党抓发展、重点抓经济、突出抓产业、关键抓项目”的时代发展理念，立足投资拉动占主导地位的阶段性特征，优化投资结构，加快建设有支撑作用的产业体系。

　　深入开展“346”攻坚活动。落实加快产业发展的“决定”，突出大项目建设，强化领导带头、分层推进、集中会办和跟踪问效，重点抓好红星乳业等绿色有机食品加工项目、恒丰6万吨特种纸等工业项目、荒沟抽水蓄能电站等新能源项目、万达广场等现代服务业项目，确保“动工之春”开工3000万元以上项目400个、产业项目个数比重达到60%以上。以产业项目攻坚为牵动，进一步扩大基础设施建设，加大棚户区改造力度，全面增强投资拉动。

　　提升园区承载功能。完善重点园区规划和产业定位，突出园区和产业特色，加快形成分工协作、错位发展的园区体系。加快基础设施、配套设施和公共服务平台建设，提高园区承载力和吸引力。在重点园区建立土地、房屋征收与土地收储一体化的净地形成机制，完善土地经营机制和出让机制，提高用地保障能力和效益。牡丹江国家经济技术开发区要加速推进中俄绿色食品产业园、生物科技园建设，全口径投资增长40%以上，进一步发挥全市经济发展的龙头作用。

　　加大招商和对上争取力度。突出大项目和重点产业，围绕重点区域和国内外强企，实施定向招商、定点“爆破”，深入开展产业存量招商、补链招商、增量招商、龙头招商，力争引进一批战略投资者、建设一批有牵动力的大项目。创新招商方式和机制，强化要素招商、企业招商、商会招商、中介招商，完善重大招商项目跟踪落实机制，全年招商引资增长20%。围绕“五大规划”的实施，创建对上争取的动员和组织机制，全市对上争取发展资金达到150亿元。

　　(二)推动对俄合作转型升级，增创沿边开放新优势。落实《黑龙江和内蒙古东北部地区沿边开发开放规划》，加速中俄地区友好合作示范城市建设，加强绥芬河、东宁口岸与中心城市、内陆县(市)互动合作，增强牡绥地区在新一轮沿边开放中的先导作用。

　　加强口岸通道建设。推进东宁口岸界河桥改造和小车通关，逐步提升口岸能级。密切与俄罗斯海参崴港、朝鲜罗津港和国内大连港、丹东港、满洲里口岸合作，支持国内企业合作开发俄远东港口，促进陆海联运大通道常态化运营，扩大丹东港集团物流规模，推进东—乌铁路对接和穆棱铁海联运项目口岸功能内移，积极谋划引进俄罗斯宽轨，在全省建设通达出海口、辐射东北亚的国际经贸大通道中，发挥更重要的作用。

　　做强开放升级载体。促进绥芬河综合保税区保税政策向内陆县(市)区延伸，整合华晟物流和源丰物流等海关特殊监管资源，支持穆棱开发区创建国家级边境贸易合作区，鼓励境内重点园区引进大型加工贸易企业，争取“属地申报、口岸放行”试点，探索在市域海关监管区域创建中俄自由贸易园(港)区。推进华信、华宇、龙跃园区晋升国家级境外园区，支持境外园区吸引大企业、大公司和高新技术企业入驻，打造“中国企业走出去”和“俄罗斯资源能源技术引进来”的双向平台。

　　壮大外向产业体系。推进“出口抓加工、进口抓落地、贸易抓转型、合作抓延伸”，提高外经贸对本地经济的贡献率。做大做强企业主体，重点培育超亿美元贸易企业和千万美元加工企业，支持中小企业建立对俄经贸合作联盟，新引进外贸企业12户。优化贸易结构，发展加工贸易、服务贸易，大力培育和支持跨境电子商务，发展跨境甩挂运输。壮大拉长跨境产业链条，以林木、油气、矿产资源和化工产品为重点，发展壮大进口加工；以电子产品、轻工商品、农副产品和建材为重点，加强加快出口加工；以发展对俄煤炭、原油、木材等资源能源深加工，开展生物工程、装备制造、新材料等高新技术领域合作为重点，拓展对俄合作领域。全市进口资源落地加工和地产品出口比重分别达到49%和15%。

　　深化对外交流合作。落实与俄罗斯远东地区城市友好交流计划，完善中俄地方政府间互访机制，强化驻海参崴商务联络处功能，打造中俄合作信息交流平台。把握中俄互办“青年友好交流年”契机，加强文教卫体等领域交流，争创中俄日韩经贸及投资合作论坛。放大绥芬河卢布使用试点效应，探索建立对俄结算中心，简化对外投资审批程序，优化中俄合作服务环境。加强与俄滨海边疆区和远东发展部的沟通，更好地融入中俄自由贸易区发展战略。

　　(三)壮大精品和品牌农业，拓展“三农”发展新空间。抢抓全省“两大平原”现代农业综合配套改革机遇，做好“转、水、林、优”四篇文章，加速特色精品农业向集约化、高效化、现代化迈进。

　　做强特色产业体系。以“一带六区”为重点，加速粮菌、粮菜、粮牧“三个转型换位”，全市新增棚室蔬菜4000亩，新增食用菌标准化园区30个、食用菌规模突破40亿袋(块)，畜牧业规模养殖比重达到75%以上，农产品出口基地达到34.5万亩。按照产业化理念，抓好皓月、响水等重点龙头和红星有机奶源等重点基地建设，规模以上龙头企业达到185户，绿色有机食品认证面积达到450万亩以上，创建国家级大型食用菌绿色食品原料基地。推进质量、市场、品牌建设，加快建立农产品产地准出管理制度，发展农产品期货市场和电子商务，拓展雨润、源丰等批发大市场物流集散功能，整合做强响水米、食用菌等地域品牌，推进特色产品向优质、高端、高效发展，加快绿色有机食品之都建设步伐，全市绿色有机产品总数达到190个以上。

　　壮大新型经营主体。做好登记管理，赋予家庭农场、专业大户市场法人主体地位，建设100个示范家庭农场，带动农业家庭经营向规模化方向发展。开展合作社规范化专项行动，提高农业合作经营规模，合作社总数达到1500个。发展壮大庄园经济，吸引社会工商资本投资农业项目，打造特色农业综合体。开展土地承包经营权资本量化和土地规模经营试点，推进土地承包权向新型经营主体集中，全市土地规模经营面积达到65万亩。

　　拓展农民增收渠道。加快农村产权确权登记，开展农村集体资产股份化改造试点和产权抵押、担保、转让试点，增加农民财产性收入。支持农民发展林下经济、旅游农业和水产养殖等接续产业，推进城郊农业转型升级，增加农民经营性收入。鼓励转移就业，支持返乡创业，拓展农民非农就业空间，增加农民工资性收入。落实强农惠农富农政策，增加农民转移性收入。

　　(四)深化“工业立市”战略，构建现代工业新体系。以实施“全国老工业基地调整改造规划”为契机，提升传统产业，壮大新兴产业，增强创新驱动，推动统筹发展，打造牡丹江工业经济升级版。

　　推进产业集群。以整机化、系列化、品牌化为方向，抓好天合石油钻井工程等重点项目建设，改造提升林纸加工、煤化石化、能源矿产等传统优势产业。整合域内资源，加大支持力度，推进烟草加工业集约发展，加快石油装备制造业国际化步伐。把握政策导向和市场需求，加快友搏制药产业园、黑宝药业产业链扩能升级等新兴项目建设，发展壮大绿色有机食品加工和生物医药产业，着力培育电子信息、新材料和节能环保产业，加速打造50亿级龙头企业和百亿级产业集群。

　　强化创新驱动。突出企业主体地位，发挥十大产业联盟、十大创新服务平台作用，强化中俄科技信息产业园孵化中心功能，启动北理工(牡丹江)成果转化中心，深化与中科院、工信部军工七院校等院校合作，新建10个企业技术研发中心、10个科技创新团队、10户科技创新型骨干企业，加快培育信息化建设示范企业，推进“两化”融合示范工程，支持企业开展电子商务，高新技术产业产值增长16%以上。

　　加强工业调控和服务。完善监测指导，强化工业大运行协调机制，增强煤、水、电、汽、运等要素保障。支持首控、佳通、富通等企业裂变升级，推进煤化、焦化和制药企业改制重组，做好集体企业改制工作。实施“小巨人”企业培育工程和小微企业梯次成长工程，完善中小微企业公共服务体系，激发全民创业活力，发展壮大非公经济和混合所有制经济，全市新增规模以上企业40户。

　　统筹区域经济发展。强化市县区一体化发展理念，优化全市产业布局，打破区域壁垒，鼓励飞地经济，促进区域经济协调发展。进一步落实开发区市级管理权限，引导政策、资金、项目向开发区集中，加快牡丹江国家经济技术开发区发展；建立市区联动的工业管理机制，推动市区工业提速增效；打好市本级财源提升工程攻坚战，提升市本级工业和财政比重。支持县(市)立足比较优势，培育支柱产业，壮大特色经济，保持县域经济全省领先水平。支持东宁和林口推进县改市。

　　(五)深化“贸旅牵动”战略，推动服务业实现新提升。全面推进“十大中心”建设，突出发展商贸、旅游、金融等优势产业和新兴服务业，加速构建区域性、国际化消费中心城市。

　　增强商贸和流通产业辐射力。加快城市商圈建设和江南商业配套，优化商业网点结构和布局，重点推进大商集团江南购物中心、绿地CBD、五洲国际商贸博览城等商业综合体、亿丰国际汽车城等专业市场、朝鲜民俗风情街等特色商街建设。发展壮大物流产业，推进交通枢纽国际物流中心、开发区现代物流园、东宁国际物流园等重点项目建设，培育华晟、牡达等10户骨干企业，谋划国际物流中心项目，进一步提升哈牡绥东国际物流园和林口、宁安、海林物流园区功能。全市商业营业面积、物流业增加值分别增长5%和10%以上。

　　加快旅游业向四季型国际化转变。推进镜泊湖北门综合体、莲花新镇等重点项目建设，提高景区景点承载能力。举办牡丹江旅游节，办好冬捕节、秋冬旅游文化节等品牌活动，深化与东北三省重点城市合作，开通至俄日韩旅游线路，推广健康游、自驾游等旅游产品，丰富四季型、国际化特色旅游产品体系，提升旅游影响力。实施“智慧旅游”工程，推进旅游信息化“一平台三体系”建设，促进景区景点互动联动。理顺镜泊湖管理体制，深化“旅游优质服务年”活动。全市接待境内外游客人数和旅游业总收入均增长19%以上。

　　提升金融产业实力和服务能力。深化“金融强市”战略，加快地方金融改革，完善地方金融机构监管，增强市融资服务中心功能，建设民间投融资服务中心。大力发展地方金融机构，推进市、县(市)两级农信社改制农村商业银行，建立农林商品交易中心，新建地方金融机构8家以上。支持新创新与金融机构合作建设产业投资基金，放大放活公积金支持保障房建设功能。推进具备条件的企业通过私募债等方式直接融资，加快友搏药业、天合石油、富通空调上市进程。加快农村金融创新，探索农村土地经营权、林权质押融资。开展“中小企业融资服务年”活动，引导金融机构支持重点项目建设和中小企业发展，域内金融机构存贷比提高5个百分点。加快社会诚信体系建设，严厉打击恶意逃废银行债务，提高金融风险防控水平，优化金融生态环境，争建全省金融创新试验区。

　　大力发展新兴服务业。出台会展业发展规划、规范会展业管理办法，加速会展业市场化、品牌化、国际化步伐，办好第六届中国牡丹江黑木耳节等重点展会，新谋划省级以上展会10个。支持社会资本兴办科技信息咨询中介服务机构。加快三道关休闲养老社区、牡丹峰养生文化村等养老产业项目建设，满足域内外多样化、高层次养老需求。

　　(六)加快新型城镇化建设，催生持续发展新动力。按照以人为本、科学布局、绿色发展、文化传承的新要求，加快“1核5城18镇100区”新型城镇化体系建设，凸显牡丹江在哈大齐牡城市群和沿边开放城镇带的地位，凸显城镇化拉动发展最大潜力的作用。

　　强化规划引领和交通建设。适应牡海宁同城化需要，突出全域规划理念，大手笔、高质量进行城市总规修改，抓好中心城市道路交通、城区水体利用等23个专项规划编制，推进新型城镇化规划编制。推进交通信息化建设，加快牡绥铁路扩能改造、哈牡高铁和牡海、牡宁城际公路等重点项目建设，推进机场迁建，畅通城际快速通道和出境出海通道。

　　提升中心城市功能。按照“五城同创”要求，加强城市建设和管理。加快市政基础建设，高标准推进世行贷款“三横两纵一慢行”项目建设，加快西十一跨铁拓宽、新安街西延、黄花路西延和东地明街至富江路段拓宽改造，打通3条断头路，整修20条巷路，翻建加固6座危旧桥梁，青梅大桥实现竣工通车；优化市区公交网络，建设和设置公共停车泊位1000个，更新环保型公交车200辆；改进供气供热工作，新增燃气用户2万户，改造供热老旧管网40公里。加强城市精细管理，推进高寒城市智能公交系统、物流运输智能服务系统建设，启动智慧城管系统一期试点；深入实施城市管理“363”工程，提升城市管理标准和水平；开展城管志愿者活动，动员社会力量管理城市，形成“人民城市人民爱、人民城市人民建、人民城市人民管、人民城市人民赞”的良好氛围。

　　提升小城镇承载力和吸引力。推广“5+1”建设模式，健全完善与森工农垦合作共建机制，发挥宁安响水“两化”示范引领作用，加快基础建设、棚户区改造和产业建设，完善综合服务功能，年内城关镇、重点镇完成170万平方米棚户区改造任务，18个重点镇每个完成投资3000万元，33个新型农村社区每个完成投资500万元。加快建立城乡统筹发展的体制机制，全面放开中心城市、城关镇和建制镇落户限制，制定优惠政策促进劳动密集型产业发展，把进城落户农民纳入城镇住房保障体系，让进城农民同等享受教育、医疗等基本公共服务，有序推进农业转移人口市民化。

　　扎实推进新农村建设。科学规划建设新型农村社区，加强农业农村基础设施和公共服务体系建设，完善治理机制，促进公共服务均等化。实施新农村美好家园和美丽乡村创建工程，发挥穆棱保安新村、宁安江西村等典型示范作用，加快建设G10、G11沿线美丽乡村示范带，力争建设10个省级环境整治先进村、20个美丽乡村精品村，打造乡愁可寄、生活幸福的美好家园。

　　(七)大力弘扬地域特色文化，打造加快发展新引擎。找准文化助力发展的切入点，挖掘特色文化，开发特色资源，打造特色品牌，促进文化大发展，提升城市软实力。

　　加强地域文化保护和传承。坚持在保护中开发、在开发中传承，深入研究牡丹江地域文化渊源，挖掘以渤海国、宁古塔为代表的历史文化，以林海雪原、八女英烈为代表的红色文化，以少数民族为代表的民俗文化等资源，打造地域特色文化品牌。加强文化遗产保护利用和非物质文化遗产保护开发，加快横道河子俄罗斯老街、牡丹江边墙等重点文物保护项目建设，提升靺鞨绣、蚕翼绣 、萨满祭祀、朝鲜族花甲礼等非遗项目的知名度和影响力。站在东北亚乃至更高层面，深入挖掘渤海国文化底蕴，做好渤海国文脉、文艺、文字、文物的研究收集、整理传承，推进渤海国遗址公园和文化产业园建设。依托丰厚的文化底蕴和高校资源，大力培养引进文化领军人物和专业人才，为地域文化传承发展奠定坚实基础。

　　加快发展文化产业。发挥独具特色的历史文化、生态文化、民俗文化、红色文化和口岸文化等资源优势，推动文化与旅游、信息、建筑、农业等领域融合发展，培育新兴文化业态和消费市场。围绕壮大文化旅游、新闻出版、影视动漫、数字信息、演艺娱乐、节庆会展、工艺美术、教育培训等八个主导产业，加速推进文化创意产业园区、国家级民族文化保护园区等园区项目，俄罗斯风情街、爱民生态农业观光园等产业项目，打造一批国家级和省级文化产业示范基地和园区。强力推进文化旅游发展，把湖、林、雪、边、俗、特、红等特色优势，转化为提升经济实力的增长点。

　　大力推进公共文化建设。放大国家级公共文化服务体系示范区综合效应，加快牡丹江文博馆等文化设施建设，完善公共设施网络体系。推进牡丹江文艺精品创作和传承，深入开展群众性文化活动，丰富群众精神文化生活。推进农村数字电影服务等惠民工程，建设全市公共文化数字共享平台，实现市、县、乡、村文化数字资源网络全覆盖。发挥中俄文化交流中心、牡丹江旅游节等载体作用，开展一批国际文化交流合作活动，深化对外人文交流，加强与俄罗斯、韩国、香港、台湾等国家和地区的文化互动合作。

　　(八)切实增进人民福祉，集聚跨越发展新能量。坚持民生优先，加快落实“和谐幸福50条”，办好28件利民实事，让发展成果更多更公平惠及全市人民，激发人民群众推进跨越发展的内在激情。

　　抓好创业就业工作。落实城乡一体化就业扶持政策，发放小额创业贷款1.5亿元，扶持自主创业5000人，争创国家级创业型城市。做好残疾人、大学生、退役军人、“4050”等重点人群就业工作，开发公益性岗位2000个，城镇新增就业6万人，城镇登记失业率控制在4%以内。

　　建设覆盖城乡、均等普惠的社保体系。建立城镇居民大病补充医疗保险制度，强化城乡养老、医保制度衔接，提高“五项”社会保险、新农合和城乡低保、“五保”待遇标准，城镇居民医保最高支付限额提高到8万元，城市“三无”、农村“五保”人员医疗报销比例达到95%。90%社区开展居家养老服务。做大“爱心帮扶基金”规模，加快救济救助常态化。

　　推进保障性住房建设。加快推进重点棚改项目，开工建设1500套公租房和廉租房，整治老旧小区庭院400万平方米，发放廉租住房补贴3500万元，改造农危房5000户，让群众住得更加放心、更加公平。

　　加快发展社会事业。推动教育均衡发展，完成15所义务教育学校标准化建设，改造7所农村薄弱学校、31所火炉取暖学校，加速推进国家示范性综合实践基地建设。支持省属高校做大做强，加快牡丹江大学升本，探索与俄韩合作办学。健全城乡卫生服务体系，促进公共卫生服务均等化，以标准化建设为契机提升医疗装备水平和管理水平，新建江南医院，鼓励医疗专家进社区，实现远程会诊全覆盖。推进东北亚体育中心建设，抓好全民健身活动。深化幸福家庭创建，切实维护妇女儿童合法权益。

　　加强和创新社会治理。深入实施“3321”工程，以安全、公平、关爱、强基为着力点，在建设“天网地格”、实施“三调联动”、创新社区体制、培育社会组织等方面探索先行，创新社会治理体系。稳步推进社区管理体制改革，新建2个城市千米精品示范社区、15个城市达标社区，推进33个新型农村社区建设。强化保供稳价和食药安全监管，启动建设肉菜流通追溯系统，开展“校园绿盾工程”，打造全省食品药品安全放心城市。加强日常监管和专项整治，强化安全生产责任落实，坚决遏制重特大事故发生。全面加强信访工作，推进阳光信访和联合接访，全市信访积案下降30%。突出构建公安“十大体系”，深入开展“三大行动”，严厉打击违法犯罪活动，建设平安牡丹江。

　　(九)加强生态文明建设，迈出绿色低碳循环发展新步伐。抓住实施“全国资源型城市可持续发展规划”机遇，坚持源头严防、过程严管、后果严惩，倡导绿色发展理念，保护好、利用好牡丹江的绿水青山。

　　加强生态保护和建设。推进城市生态园林建设，加快推进沿江景观带、江心岛和跨江步行景观桥建设，新建改造3个公园广场、13个街头游园，市区新增绿地面积50万平方米。提升牡丹江、穆棱河、绥芬河三大流域水质，推进镜泊湖、莲花湖、城乡湖泡及小流域综合治理，抓好奋斗水库等水源工程，治理水土流失36万亩。推进退耕还林和三北五期天然林保护工程，全市退耕还林10万亩、育林造林15万亩。积极开展生态镇村创建活动。

　　狠抓节能减排和综合治理。实施工程措施和管理措施两手抓，落实企业节能减排责任主体，严格执行项目准入、运行标准和节能减排目标责任制。实施二发电等8个大气减排项目，完成灵泰药业等10个污水治理项目，国家和省控重点污染源企业污防设施运行率达到100%。加快新能源开发利用，鼓励发展循环经济，工业固废综合利用率达到89%，规模以上工业万元增加值综合能耗下降10%以上。加强市区大气污染治理，淘汰取缔黄标车800辆，拆除小烟囱1.5万个，开展PM2.5空气环境质量监测。推进污水和垃圾治理，污水处理二期工程、生活垃圾无害化处理二期工程投入运行。

　　三、加强政府改革和建设

　　按照中央全面深化改革“决定”和省委、市委的战略部署，统筹推进各项改革，着力建设法治型政府、创新型政府、服务型政府和责任型政府，为我市经济社会跨越争先提供坚强保障。

　　加快推进政府机构改革。启动新一轮政府机构改革，推进卫生、人口计生部门重组，完成县(市)区食品药品监管部门机构、职责、编制调整工作。按照精简统一效能原则，探索大部门制改革，探索企业、社会组织和事业单位登记制度改革，推进产权登记制度等改革政策落实。严格控制机构编制，财政供养人员只减不增。

　　深化行政管理服务改革。正确处理政府与市场的关系，政府该管的事管起来、管到位，更好地担负起市场监管、公共服务、社会管理、环境保护的责任。按层次承接好中央和省下放的行政审批事项，取缔中央和省明令取消的审批事项，取缔违规设立的审批事项，取缔保留事项中的违规违法前置条件。推进简政放权，理顺市与县(市)区事权关系，继续推动向重点镇下放经济社会事权。以电子政务建设为主线，推进行政审批集成化，实施“一图制”、“一体式”、“一表制”审批；推进行政事业收费透明化，实施“一卡制”收费；推进行政执法集中化，实施“一队式”执法；推进行政服务便利化，实行网上审批、全程代办、送照上门；推进行政监督民主化，强化监察、审计和社会全方位监督，加快建设“五个平台一条线”集成式政府管理服务体系。

　　稳步推进事业单位分类改革。按照政事分开、事企分开原则，提高政府公共服务水平。在明确事业单位分类的基础上，推进承担行政职能事业单位向行政单位转并；公益性事业单位加快去行政化进程，探索建立法人治理结构；有社会化特点的公益性事业单位，引入竞争机制，加大政府购买服务力度；加快经营性事业单位向企业转型。

　　深化财税体制改革。改进预算管理，完善以支出为重点的一般性和基金预算编制工作，提高国有资本经营预算编制水平，试编政府债务预算，加强预算系统性、规范性。拓宽投融资渠道，统筹资金使用。加强财政支出管理，优化支出结构，压缩“三公经费”和行政事业性项目支出5%。

　　加强民主法制建设。完善和创新科学民主决策制度，大力推进政务公开，开门办政府，探索人大代表、政协委员、百姓及企业等利益相关方代表参与政府决策的机制，问政于民、问需于民、问计于民，确保决策科学合理、顺应民意。严格依法行政，依照法定权限和程序行使权力、履行职责，强化执法监督和行政问责。全面实施“六五”普法规划，进一步推进行政复议委员会试点改革。自觉接受人大法律监督、政协民主监督和公众社会监督，广泛听取民主党派、工商联、无党派人士、社会各界意见和建议，认真办理、及时回复人大代表建议和政协委员提案。全面完成第三次全国经济普查任务。

　　加强勤政廉政建设。扎实开展党的群众路线教育实践活动，深化“接地气、连民心”活动，大力倡导“三实两创”，在求实务实落实、创新创一流上狠下功夫，按照市委的要求，不跑调、不变调、不起高调，不搞假大空、不搞概念化、不搞形象工程，不搞水中月镜中花、看上去很美、实则无用的项目，着力解决“四风”问题。深入开展“五治五提”和“万人评百科”活动，发挥“大督查”机制和目标管理的作用，抓规范、重标准、讲担当、提效率，增强政府工作的系统性和实效性。继续深化对办公用房、公务接待、因公出国、会议活动、公务用车等专项治理，建立厉行节约反对浪费长效机制。认真落实党风廉政建设责任制，继续加强廉政风险防控，完善行政权力运行监督机制，把廉政建设的要求落实到政府工作的各个环节。加强重大项目决策、重点领域事项和重要资金使用审计，坚决纠正损害群众利益的不正之风，严肃查处行政不作为、乱作为等问题，做到干部清廉、政府清正、政治清明。

　　各位代表，我们正站在历史新起点，加快牡丹江发展的使命无比光荣，人民群众寄予的期望重如泰山。让我们在市委的坚强领导下，在人大政协的支持帮助下，在全市人民的共同努力下，振作精神、真抓实干、凝心聚力、狠抓落实，全力开创牡丹江改革发展新局面!
